# Analgesic and Local Anesthetic Effects of a Clove‐Derived Injectable Formulation via Modulation of Oxidative Stress and Nociceptive Signaling

**DOI:** 10.1155/prm/5004039

**Published:** 2026-09-20

**Authors:** Hyunseong Kim, Jin Young Hong, Junseon Lee, Hyun Kim, Changhwan Yeo, Wan-Jin Jeon, Yoon Jae Lee, Seung Ho Baek, In-Hyuk Ha

**Affiliations:** ^1^ Jaseng Spine and Joint Research Institute, Jaseng Medical Foundation, Gangnamdae-ro 540, Seoul 135-896, South Korea, jaseng.org; ^2^ College of Korean Medicine, Dongguk University, 32 Dongguk‐ro, Ilsandong‐gu Goyang‐si, 10326, South Korea, dongguk.edu

## Abstract

The major bioactive constituent of *Syzygium aromaticum* (clove), eugenol, has been reported to possess analgesic and local anesthetic properties. However, direct evidence regarding the local anesthetic and antinociceptive effects of clove extract itself or its injectable formulations remains limited. Therefore, this study evaluated the neuroprotective, antinociceptive, and local anesthetic‐like effects of *S. aromaticum* pharmacopuncture (SAP) using *in vitro* and *in vivo* models, with comparative assessment against lidocaine. Primary dorsal root ganglion (DRG) neurons were exposed to H_2_O_2_‐induced oxidative stress and treated with SAP. Cell viability, neurite outgrowth, intracellular reactive oxygen species (ROS), and pain‐related markers, including calcitonin gene‐related peptide (CGRP), isolectin B4 (IB4), and substance P, were evaluated. *In vivo* analgesic and anesthetic effects, including CFA‐induced inflammatory pain, were assessed using tail flick, von Frey, and hot plate tests in rat models. SAP significantly improved cell viability and promoted neurite outgrowth while attenuating oxidative stress‐induced cellular damage. Additionally, SAP markedly suppressed intracellular ROS levels and decreased the expression of pain‐related markers, including CGRP, IB4, and substance P. *In vivo*, high‐dose SAP significantly increased tail flick latency, reaching cutoff levels comparable to those of 0.4% lidocaine. Furthermore, SAP attenuated mechanical hypersensitivity in the CFA‐induced inflammatory pain model and reduced microglial activation, inflammatory mediators (cyclooxygenase‐2, interleukin‐1β), and nociceptive marker expression in DRG and spinal cord tissues. These findings provide experimental evidence supporting the antinociceptive and local anesthetic‐like potential of SAP and highlight the involvement of oxidative stress modulation and nociceptive signaling in its pharmacological actions.

## 1. Introduction

Effective pain management remains a major clinical challenge, particularly in conditions where nociception is accompanied by inflammation and neural tissue damage [1]. Acute and chronic pain states are often driven not only by peripheral nerve excitation but also by oxidative stress, inflammatory mediator release, and sensitization within the peripheral and central nervous systems [2, 3]. For this reason, therapeutic strategies that solely suppress nerve conduction may be insufficient to provide sustained or comprehensive symptom relief. Local anesthetics such as lidocaine are commonly used in clinical practice because of their rapid onset and reliable nerve‐blocking properties [4]. However, conventional anesthetics are primarily designed to interrupt action potential propagation and generally have limited influence on inflammatory cascades, oxidative injury, or tissue recovery [5–7]. In inflammatory pain conditions, persistent cytokine signaling, glial activation, and nociceptor sensitization may continue even after transient anesthetic effects subside [8, 9]. Accordingly, there is growing demand for agents that combine anesthetic efficacy with broader biological actions relevant to pain pathophysiology [10]. Medicinal plants have attracted increasing attention as sources of multimodal analgesic compounds. Herbal materials often contain structurally diverse phytochemicals capable of modulating multiple molecular targets simultaneously, including ion channels, inflammatory enzymes, redox pathways, and neuroimmune signaling networks [11, 12]. Such pleiotropic actions may offer advantages in complex pain disorders where multiple mechanisms coexist [13, 14].

*Syzygium aromaticum* has long been traditionally used for the relief of toothache and painful inflammatory conditions [15]. Its principal bioactive constituent, eugenol, has been reported to possess analgesic and local anesthetic properties, partly through inhibition of voltage‐gated sodium channels (VGSCs) [16]. Recent electrophysiological studies have further shown that eugenol and lidocaine inhibit sodium channels through related but mechanistically distinct pathways, supporting the potential of clove‐derived compounds as alternative anesthetic agents [16, 17]. However, these findings have largely been limited to isolated eugenol rather than clove extract itself. Accordingly, the application of these pharmacological properties to standardized injectable formulations remains insufficiently investigated. In particular, limited evidence is available regarding whether a clove‐derived pharmacopuncture preparation can exert clinically relevant local anesthetic effects while simultaneously modulating oxidative neuronal injury, nociceptive signaling, and inflammatory pain mechanisms [18]. Clarifying these effects is essential for determining the therapeutic value of such formulations beyond traditional or empirical use. Therefore, the present study investigated the pharmacological effects of *S. aromaticum* pharmacopuncture (SAP) using complementary *in vitro* and *in vivo* approaches. Primary dorsal root ganglion (DRG) neurons were used to evaluate neuronal protection under oxidative stress and changes in pain‐related molecular markers. In parallel, rat models of inflammatory pain were used to evaluate the local anesthetic and analgesic effects of SAP, which were comparatively assessed against those of lidocaine. Through these approaches, we sought to characterize the neuroprotective and pain‐modulating properties of SAP, including its potential local anesthetic and antinociceptive activities.

## 2. Materials and Methods

### 2.1. Preparation and Sterilization of SAP

SAP was manufactured at the Jaseng External Herbal Dispensary (Seoul, Korea) under Good Manufacturing Practice (GMP) conditions. Briefly, dried *S. aromaticum* (600 g) was extracted with 10 volumes of purified water at 100°C for 2 h, followed by steam distillation at 100°C for 4 h to obtain the distilled extract. The extract was subsequently cooled (0–10°C) to remove the upper oil layer and impurities, and the purification process was repeated before formulation. The resulting extract was transferred to a mixing tank, mixed for 30 min, and sequentially sterilized by double filtration through a 0.2‐μm membrane filter. The sterile filtrate was aseptically filled into 10‐mL glass vials, sealed with rubber stoppers and aluminum caps, and terminally sterilized before visual inspection and packaging. Quality control of the final formulation included evaluation of appearance, pH, density, sterility, bacterial endotoxin, container integrity, and quantitative determination of eugenol by HPLC. The SAP preparation used in this study contained 1.036 μL/mL eugenol, corresponding to an eugenol concentration of 6.875 mM in the final formulation.

### 2.2. Quantitative Analysis of Eugenol by HPLC

The eugenol content of SAP was quantified using a high‐performance liquid chromatography (HPLC) system equipped with a diode‐array detector (Agilent Technologies, Santa Clara, CA, USA). Chromatographic separation was performed on a TC‐C18(2) column (4.6 × 250 mm, 5 μm). The mobile phase consisted of water and methanol with gradient elution (0 min, 60:40; 10 min, 40:60; 35 min, 60:40; 40 min, 60:40, v/v) at a flow rate of 1.0 mL/min. The column oven temperature was maintained at 40°C, and the injection volume was 10 μL. Eugenol was detected at 280 nm using a diode‐array detector. Quantification was performed by comparison with an authentic eugenol reference standard. Repeatability of the SAP preparation was confirmed by six independent injections.

### 2.3. Primary Rat DRG Neurons

Primary DRG neurons were prepared from 5‐ to 6‐week‐old male Sprague–Dawley (KOATECH, Pyeongtaek, Korea) rats according to the procedures approved by the Korean Animal Ethics Committee (Approval No. JSR‐2023‐02‐002‐A‐002). After euthanasia using carbon dioxide (20%–30%/min chamber volume), cardiac perfusion was performed using cold Hank’s balanced salt solution containing heparin. The C2–L7 spinal cord segments to which the DRGs were attached were detached, and the surrounding connective tissue and neuromuscular tracts were carefully removed using a dissecting microscope. Detached DRGs were subjected to enzymatic digestion using 40 U/mL papain (Worthington, Lakewood, NJ, USA), 4 mg/mL collagenase type II (Worthington), and 4.66 mg/mL dispase (Roche, Basel, Switzerland), followed by gentle pulverization to obtain single‐cell suspensions. To minimize non‐neuronal adhesion, cells were precultured and transferred to 20 μg/mL poly‐D‐lysine and 10 μg/mL laminin‐coated plates, then cultured in Neurobasal medium supplemented with 2% B27 (Thermo Fisher Scientific, Waltham, MA, USA), 1% GlutaMAX (Thermo Fisher Scientific), 1% penicillin/streptomycin (Thermo Fisher Scientific), and 10 ng/mL brain‐derived neurotrophic factor (Thermo Fisher Scientific). All procedures were performed under sterile conditions to optimize neuronal viability and downstream analyses. Detailed experimental parameters, including enzyme concentrations and incubation conditions, were based on previously published methodology [19].

### 2.4. SAP Treatment and Experimental Groups

Primary DRG neurons were subjected to short‐term and long‐term culture protocols. For the short‐term protocol, freshly isolated DRG neurons were cultured for 2 days, and treatments were initiated on Day 3 *in vitro*. For the long‐term protocol, neurons were maintained for 10 days, and treatments were initiated on Day 11 *in vitro*. SAP stock solution (6.875 mM) was diluted in culture medium to final concentrations of 10, 25, 50, 100, 250, and 500 μM. Cells were treated with SAP alone or co‐treated with 100 μM hydrogen peroxide (H_2_O_2_) to induce oxidative stress. All treatments were maintained for 24 h prior to analysis. Based on the concentration screening results, 25, 50, and 100 μM SAP were selected for subsequent experiments. The experimental groups were as follows: (1) blank, untreated control; (2) H_2_O_2_, H_2_O_2_‐treated group; (3) SAP25, H_2_O_2_ + SAP 25 μM; (4) SAP50, H_2_O_2_ + SAP 50 μM; and (5) SAP100, H_2_O_2_ + SAP 100 μM. Following treatment, neuronal viability, neurite outgrowth, intracellular reactive oxygen species (ROS), and pain‐related molecular markers were assessed.

### 2.5. Cell Viability Assay

DRG neuronal viability after SAP and/or H_2_O_2_ treatment was evaluated using a Cell Counting Kit‐8 assay (CCK‐8; Dojindo, Kumamoto, Japan). Cells were seeded in 96‐well plates and cultured under standard conditions (37°C, 5% CO_2_). Following treatment for 24 h, 10 μL of CCK‐8 reagent was added to each well and incubated for 4 h. Absorbance was measured at 450 nm using a microplate reader (Epoch, BioTek Instruments, Winooski, VT, USA). Cell viability was expressed as a percentage relative to the untreated control group.

### 2.6. Immunocytochemistry

Primary cultured DRG neurons were fixed with 4% paraformaldehyde (PFA) for 30 min and washed with phosphate‐buffered saline (PBS). Cells were permeabilized with 0.2% Triton X‐100 for 10 min and blocked with 2% normal goat serum for 1 h at room temperature. Samples were then incubated overnight at 4°C with primary antibodies against Tuj1 (R&D Systems, Minneapolis, MN, USA), IB4 (Sigma‐Aldrich, St. Louis, MO, USA), CGRP (Sigma‐Aldrich), and Iba1 (Cell Signaling Technology, Danvers, MA, USA). After washing, cells were incubated with fluorescein isothiocyanate‐conjugated secondary antibodies (Jackson ImmunoResearch, West Grove, PA, USA) for 2 h at room temperature in the dark. Following final washes, coverslips were mounted using fluorescence mounting medium, and images were acquired using a confocal microscope (Eclipse C2 Plus; Nikon, Tokyo, Japan) at 10× or 40× magnification.

### 2.7. Substance P Quantification by Enzyme‐Linked Immunosorbent Assay

The concentration of substance P released into the culture medium was measured using a Substance P Parameter Assay Kit (R&D Systems, Minneapolis, MN, USA) according to the manufacturer’s instructions. DRG neurons were cultured for 10 days, and on Day 11, cells were treated with SAP in the presence of H_2_O_2_ for 24 h. After treatment, culture supernatants were collected and centrifuged at 1000 × *g* for 10 min to remove debris. The clarified supernatants were subjected to ELISA analysis. Absorbance was measured at 450 nm using a microplate reader (Epoch, BioTek Instruments, Winooski, VT, USA), and substance P concentrations were determined from a standard calibration curve. All samples were analyzed in duplicate.

### 2.8. In Vivo Experimental Model

Male Sprague–Dawley rats (8 weeks old; Koatech Bio, Republic of Korea) were used for all in vivo experiments to minimize potential variability associated with estrous cycle‐related hormonal fluctuations that may affect pain sensitivity. A total of 30 rats were randomly assigned to five experimental groups (*n* = 6 per group): (1) sham, saline injection without CFA; (2) CFA, 100 μL complete Freund’s adjuvant (1 mg/mL; Sigma‐Aldrich) followed by 100 μL vehicle (sterile saline); (3) SAP100, CFA plus 100 μL SAP containing 6.875 mM eugenol; (4) SAP200, CFA plus 200 μL SAP containing 6.875 mM eugenol; and (5) lidocaine, CFA plus 0.4% lidocaine. Animals were housed under controlled conditions (25 ± 1°C, 50 ± 5% humidity, 12 h light/dark cycle) with free access to food and water and allowed to acclimate before experimentation. All animal use and care followed the guidelines approved by the Jaseng Animal Care and Use Committee (JSR‐2025‐01‐001‐A‐001). Behavioral assessments and quantitative outcome analyses were performed by investigators blinded to the group assignments. For induction of the inflammatory pain model, 100 μL of CFA (1 mg/mL) was first loaded into a 1‐mL syringe, followed sequentially by 100 or 200 μL of SAP containing 6.875 mM eugenol, without premixing. The solutions were administered consecutively through a single subcutaneous injection into the plantar surface of the hind paw. This procedure enabled simultaneous CFA induction and SAP administration while minimizing repeated needle insertion and additional tissue injury.

### 2.9. Tail Flick Test

Local anesthetic activity of SAP was evaluated using the tail flick test. A ceramic heating lamp was preheated for at least 20 min before testing. Rats were placed in a restrainer and allowed to acclimate for 10 min prior to the experiment. Test solutions were injected subcutaneously at the bilateral ventral midline adjacent to the sixth coccygeal vertebral region of the tail, with half of the total volume administered to each side. The experimental groups were as follows: (1) sham, normal saline (100 μL total); (2) SAP50, 1:1 diluted SAP (100 μL total); (3) SAP100, undiluted SAP (100 μL total); (4) SAP200, undiluted SAP (200 μL total); and (5) Lido, 0.4% lidocaine solution (100 μL total). Ten minutes after injection, the treated tail region was positioned over the center of the heating source. Latency to tail withdrawal (tail flick response) in response to thermal stimulation was recorded in seconds. Each animal was tested three times, and the mean value was used for statistical analysis. Preliminary optimization experiments using the same experimental procedures were performed prior to the main study to determine the appropriate thermal cutoff time and the optimal lidocaine concentration used as the positive control for the tail flick test. The results of these preliminary experiments are presented in Supporting Figure S1. A cutoff time of 8 s was applied to prevent tissue damage, and animals showing no response within 8 s were assigned the maximum latency value.

### 2.10. Hot Plate Test

Acute analgesic effects of SAP were evaluated using the hot plate test in rats with CFA‐induced inflammatory pain. The hot plate apparatus was maintained at 55°C throughout the experiment. Briefly, all test solutions were administered by subcutaneous injection into the plantar surface of the hind paw. The experimental groups consisted of (1) sham, saline‐treated control; (2) CFA; (3) SAP100, CFA + SAP 100 μL; (4) SAP200, CFA + SAP 200 μL; and (5) Lido, CFA + 0.4% lidocaine 100 μL. The diluted SAP50 group was excluded from subsequent antinociceptive tests because it showed no significant effect in the tail flick test. Thirty minutes after injection, each animal was placed on the heated surface, and the latency to a nociceptive response, defined as paw licking, paw withdrawal, or jumping, was recorded in seconds. Each animal was tested four times with an intertrial interval of at least 5 min, and the mean value was used for analysis. A cutoff time of 30 s was applied to prevent thermal injury, and animals reaching the cutoff time were assigned the maximum latency value.

### 2.11. Von Frey Test

Mechanical hypersensitivity was evaluated using an electronic von Frey apparatus in rats with CFA‐induced inflammatory pain. Before testing, each rat was placed individually in a transparent acrylic chamber with a metal mesh floor and allowed to acclimate for 20–30 min. Drug administration sites, injection procedures, and group allocation were identical to those used in the hot plate test. Baseline measurements were obtained before injection (0 min), followed by measurements at 5, 30, 60, and 120 min after injection. The paw withdrawal threshold in response to mechanical stimulation was automatically recorded using an electronic von Frey apparatus as the force (g) required to elicit paw withdrawal. Each animal was tested three times at each time point, and the mean value was used for analysis.

### 2.12. Immunohistochemistry

At the end of the experiment, rats were deeply anesthetized and transcardially perfused with PBS followed by 4% PFA. The L4–L6 spinal cord and DRG tissues were collected, postfixed in 4% PFA for 24 h, and cryoprotected in 30% sucrose for 3 days. Tissues were then sectioned at 16 μm thickness using a cryostat (CM1520, Leica Biosystems, Nussloch, Germany) and mounted onto slides. For staining, tissue sections were permeabilized with 0.2% Triton X‐100 in PBS for 10 min, except for spinal cord sections used for Iba1 staining, in which 0.3% Triton X‐100 was applied. After washing with PBS, sections were blocked with 2% normal goat serum in PBS for 1 h at room temperature. Sections were then incubated with primary antibodies diluted in blocking solution overnight at 4°C. For spinal cord tissue, sections were incubated with rabbit anti‐Iba1 antibody (1:100; Abcam, Cambridge, UK) for 72 h at 4°C. For DRG tissue, pain‐related neuronal markers were evaluated using rabbit anti‐CGRP antibody (1:200; Invitrogen, Grand Island, NY, USA), IB4 conjugate (1:200; Invitrogen), and guinea pig anti‐NeuN antibody (1:500; Synaptic Systems, Goettingen, Germany).

After washing, sections were incubated with appropriate fluorophore‐conjugated secondary antibodies for 2 h at room temperature in the dark. Sections were then washed and mounted with fluorescence mounting medium. Fluorescence images were acquired using a confocal microscope (Eclipse C2 Plus; Nikon, Tokyo, Japan) under identical exposure conditions. In spinal cord sections, microglial activation was assessed by measuring Iba1 fluorescence intensity using ImageJ software. In DRG sections, pain‐related neuronal marker expression was quantified as the percentage of CGRP/NeuN or IB4/NeuN double‐positive cells among NeuN‐positive neurons.

### 2.13. Real‐Time Polymerase Chain Reaction

Quantitative real‐time polymerase chain reaction (qPCR) was performed to measure mRNA expression. Total RNA was extracted from spinal cord tissue using the RNeasy Fibrous Tissue Mini Kit (Qiagen, Hilden, Germany) and reverse‐transcribed into cDNA using AccuPower® CycleScript™ RT PreMix (Bioneer, Korea) containing oligo(dT)20 primers. RNA purity was evaluated based on the A260/A280 absorbance ratio, and the individual values are provided in Supporting Table S3. qPCR was performed using iQ SYBR Green Supermix (Bio‐Rad, Hercules, CA, USA) on a CFX Connect Real‐Time PCR Detection System (Bio‐Rad). The thermal cycling conditions consisted of an initial denaturation at 95°C for 3 min, followed by 45 cycles of denaturation at 95°C for 15 s and annealing/extension at 60°C for 30 s. A melting curve analysis was subsequently performed to assess amplification specificity. Gene expression levels were normalized to GAPDH and expressed as fold changes relative to the CFA group. The target genes were selected to evaluate complementary aspects of pain‐related signaling, including neuronal activation (*FOS*), neuroinflammatory responses (*COX-2* and *IL-1β*), and endogenous opioid‐mediated analgesic signaling (*OPRM1*). The primer sequences used for quantitative real‐time PCR are listed in Table 1.

**TABLE 1:** Primer sequences used for real‐time PCR analysis.

Gene	5′–3′	Primer sequence
*FOS*	Forward	TGAAACCACCTCTGCATTCCA
	Reverse	GACGCCTCGCCTTCTTGAA

*COX-2*	Forward	CTCAGCCATGCAGCAAATCC
	Reverse	GGGTGGGCTTCAGCAGTAAT

*IL-1β*	Forward	TTGCTTCCAAGCCCTTGACT
	Reverse	GGTCGTCATCATCCCACGAG

*OPRM1*	Forward	CGATTCCAGAAACCACATTTCA
	Reverse	TGTTCGTGTAACCCAAAGCAAT

*GAPDH*	Forward	CCCCCAATGTATCCGTTGTG
	Reverse	TAGCCCAGGATGCCCTTTAGT

### 2.14. Statistical Analysis

Data are presented as the mean ± standard error of the mean (SEM). Statistical analyses were performed using GraphPad Prism 8 (GraphPad Software, San Diego, CA, USA). Comparisons among multiple independent groups were performed using one‐way analysis of variance (ANOVA) followed by Tukey’s multiple‐comparison test. Time‐course data from the von Frey test were analyzed using two‐way repeated‐measures ANOVA, with treatment group and time as factors, followed by an appropriate post hoc multiple‐comparison test. Statistical significance was defined as follows: ^#^ *p* < 0.05, ^##^ *p* < 0.01, ^###^ *p* < 0.001, and ^####^ *p* < 0.0001 compared with the blank or sham group; and ^∗^ *p* < 0.05, ^∗∗^ *p* < 0.01, ^∗∗∗^ *p* < 0.001, and ^∗∗∗∗^ *p* < 0.0001 compared with the H_2_O_2_ or CFA group.

## 3. Results

### 3.1. Phytochemical Characterization of SAP

To ensure the chemical consistency of the formulation used throughout this study, SAP was characterized by HPLC prior to biological evaluation. The chromatographic profile of SAP showed a major peak at a retention time corresponding to that of the authentic eugenol standard. Quantitative analysis confirmed that the SAP preparation contained 1.036 μL/mL eugenol (6.875 mM). Representative chromatograms of the reference standard and SAP are shown in Supporting Figure S2.

### 3.2. SAP Protects DRG Neurons From H_2_O_2_‐Induced Oxidative Stress and Promotes Neurite Outgrowth

**FIGURE 1:**
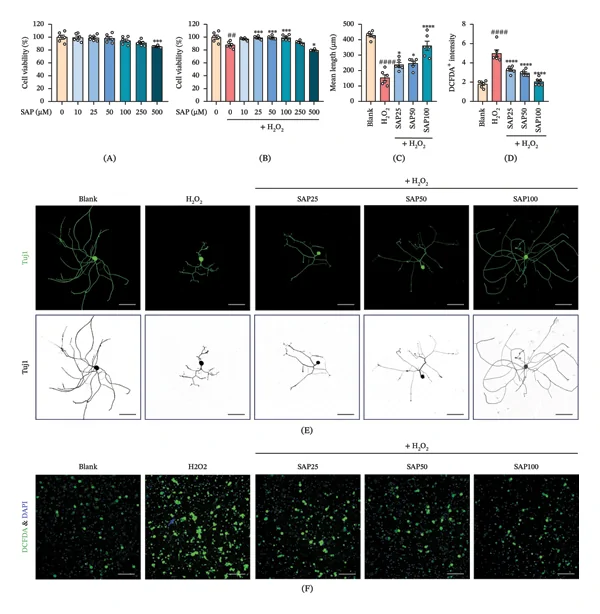
Effects of SAP on cell viability, neurite outgrowth, and ROS levels in H_2_O_2_‐treated DRG neurons. (A) Cell viability of primary DRG neurons treated with SAP alone (10–500 μM) for 24 h, as determined by CCK‐8 assay (*n* = 6). (B) Protective effects of SAP against H_2_O_2_‐induced cytotoxicity in DRG neurons, as assessed by CCK‐8 assay (*n* = 6). (C) Quantitative analysis of mean neurite length in Tuj1‐positive DRG neurons (*n* = 6). (D) Quantification of DCFDA fluorescence intensity (*n* = 6). (E) Representative immunofluorescence images of Tuj1‐stained DRG neurons showing neurite morphology in the blank, H_2_O_2_, and SAP‐treated groups. White and black scale bar = 200 μm. (F) Representative fluorescence images of DCFDA staining showing intracellular ROS levels in DRG neurons. The white scale bar = 200 μm. Data are presented as mean ± SEM. Statistical significance was analyzed by one‐way ANOVA followed by appropriate post hoc tests. ^##^ *p* < 0.01 and ^####^ *p* < 0.0001 versus blank group; ^∗^ *p* < 0.05, ^∗∗∗^ *p* < 0.001, ^∗∗∗∗^ *p* < 0.0001 versus H_2_O_2_ group.

To evaluate the cytotoxicity of SAP and determine the concentration range suitable for subsequent neuroprotection experiments in primary DRG neurons, a CCK assay was performed. Treatment with SAP alone showed a decreasing trend in cell viability with increasing concentrations; however, a statistically significant reduction was observed only at 500 μM (Figure 1A). H_2_O_2_ exposure significantly reduced cell viability compared to that in the untreated control. Co‐treatment with SAP increased cell viability in a dose‐dependent manner up to 50 μM, followed by a decreasing trend at higher concentrations. Based on these results, 25, 50, and 100 μM were selected for subsequent experiments because they represented the effective concentration range while allowing evaluation of the concentration–response relationship (Figure 1B). Neurite outgrowth was further analyzed using Tuj1 immunocytochemical staining at the single‐cell level (Figure 1C, D). At the single‐cell level in Tuj1‐stained DRG neurons, H_2_O_2_ treatment markedly reduced neurite formation and elongation compared with the blank group. Co‐treatment with SAP showed a trend toward recovery of neurite outgrowth with increasing concentrations. Quantitative analysis showed that the mean neurite length was significantly decreased by H_2_O_2_ treatment and significantly increased following SAP treatment at all tested concentrations. Intracellular ROS levels were assessed using 2′,7′‐dichlorofluorescein diacetate (DCFDA) staining (Figure 1E, F). H_2_O_2_ treatment markedly increased DCFDA fluorescence intensity in DRG neurons, whereas SAP treatment attenuated intracellular ROS levels, showing a decreasing trend with increasing concentrations.

### 3.3. SAP Reduces Nociceptive Neuron Markers and Neuropeptide Expression in DRG Neurons

**FIGURE 2:**
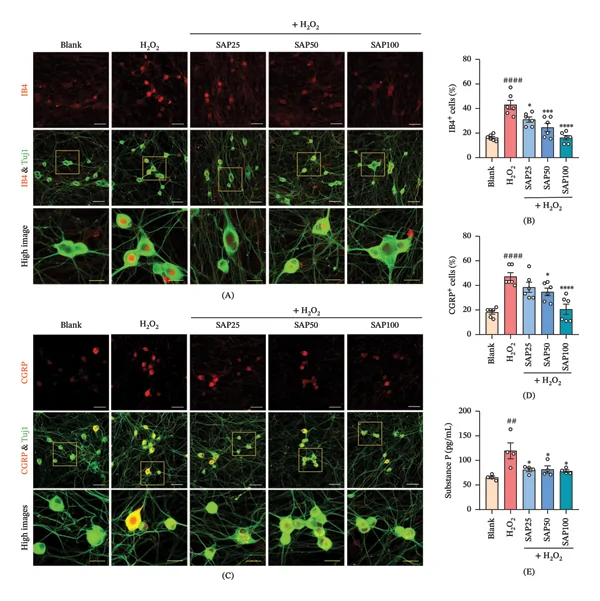
Effects of SAP on IB4, CGRP, and substance P expression in DRG neurons. (A) Representative immunofluorescence images of Tuj1/IB4‐stained DRG neurons in the blank, H_2_O_2_, and SAP‐treated groups. The white scale bar = 50 μm, and the yellow scale bar = 20 μm. (B) Quantitative analysis of the percentage of IB4/Tuj1 double‐positive neurons (*n* = 6). (C) Representative immunofluorescence images of Tuj1/CGRP‐stained DRG neurons. The white scale bar = 50 μm, and the yellow scale bar = 20 μm. (D) Quantitative analysis of the percentage of CGRP/Tuj1 double‐positive neurons (*n* = 6). (E) Substance P levels in culture supernatants measured by ELISA (*n* = 4). Data are presented as mean ± SEM. Statistical significance was analyzed by one‐way ANOVA followed by appropriate post hoc tests. ^##^ *p* < 0.01 and ^####^ *p* < 0.0001 versus blank group; ^∗^ *p* < 0.05, ^∗∗∗^ *p* < 0.001, and ^∗∗∗∗^ *p* < 0.0001 versus H_2_O_2_ group.

To further characterize the effects of SAP on pain‐related DRG neuronal markers, IB4 and CGRP immunostaining were performed. Representative immunofluorescence images of Tuj1/IB4‐stained DRG neurons showed that H_2_O_2_ treatment markedly increased IB4 immunoreactivity compared with the blank group, whereas cotreatment with SAP attenuated this increase (Figure 2A). Quantitative analysis of the percentage of IB4/Tuj1 double‐positive neurons demonstrated a significant increase in the H_2_O_2_‐treated group relative to the blank group (Figure 2B). SAP treatment significantly reduced the proportion of IB4‐positive neurons beginning at 25 μM, with further decreases observed at higher concentrations. Similarly, representative immunofluorescence images of Tuj1/CGRP‐stained DRG neurons showed that H_2_O_2_ exposure markedly increased CGRP immunoreactivity compared with the blank group, whereas co‐treatment with SAP attenuated this increase (Figure 2C). Quantitative analysis of the percentage of CGRP/Tuj1 double‐positive neurons demonstrated a significant increase in the H_2_O_2_‐treated group relative to the blank group (Figure 2D). SAP treatment significantly reduced the proportion of CGRP‐positive neurons, with significant effects observed at 50 and 100 μM. Substance P levels in the culture supernatant were further quantified by ELISA (Figure 2E). H_2_O_2_ treatment significantly increased substance P release compared with the blank group, whereas SAP co‐treatment significantly reduced substance P levels at all tested concentrations. No clear dose‐dependent trend was observed, and the mean values were comparable among the SAP‐treated groups.

### 3.4. SAP Exhibits Local Anesthetic‐like and Antinociceptive Activities In Vivo and Attenuates Nociceptive Marker Expression in DRG Tissues

**FIGURE 3:**
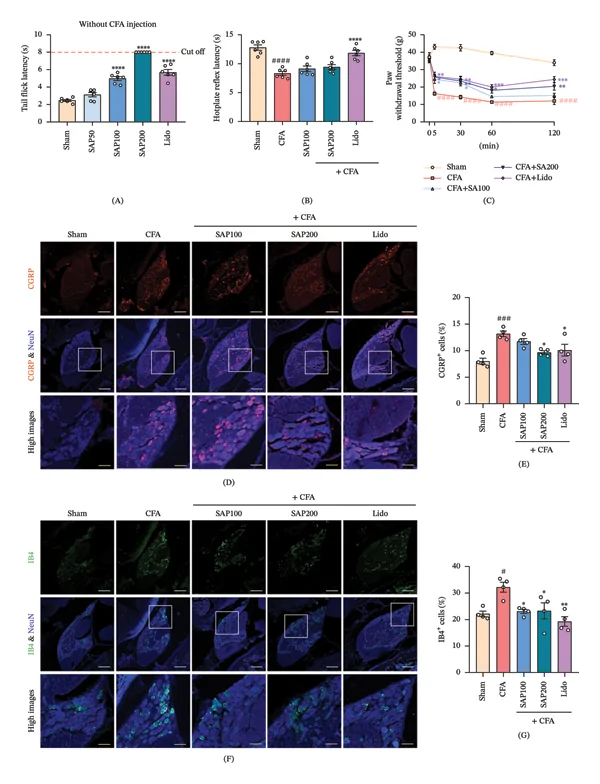
Effects of SAP on pain behaviors and DRG nociceptive markers in rats. (A) Tail flick test for evaluation of local anesthetic activity, measured 10 min after injection, with a cutoff value of 8 s (*n* = 6). (B) Hot plate test for acute thermal analgesia in CFA‐induced inflammatory pain rats (*n* = 6). (C) Paw withdrawal threshold assessed using the von Frey test at baseline (0 min), followed by measurements at 5, 30, 60, and 120 min after injection (*n* = 6). The 0‐min measurement represents the baseline value measured before injection. (D) Representative immunofluorescence images of CGRP/NeuN staining in DRG tissues. The white scale bar = 200 μm, and the yellow scale bar = 50 μm. (E) Quantification of the percentage of CGRP/NeuN double‐positive cells in DRG tissues (*n* = 4). (F) Representative immunofluorescence images of IB4/NeuN staining in DRG tissues. The white scale bar = 200 μm, and the yellow scale bar = 50 μm. (G) Quantification of the percentage of IB4/NeuN double‐positive cells in DRG tissues (*n* = 4). For B–G, all groups except the sham group received a CFA injection. Data are presented as mean ± SEM. Statistical significance was determined by one‐way or two‐way ANOVA followed by appropriate post hoc tests. ^#^ *p* < 0.05, ^###^ *p* < 0.001, and ^####^ *p* < 0.0001 versus sham group; ^∗^ *p* < 0.05, ^∗∗^ *p* < 0.01, ^∗∗∗^ *p* < 0.001, ^∗∗∗∗^ *p* < 0.0001 versus CFA group.

Tail flick latency reflects nociceptive responsiveness following local administration, with longer response times indicating greater antinociceptive activity (Figure 3A). The sham group showed a baseline response latency of approximately 2 s. SAP50 did not produce a significant change in tail flick latency compared with the sham group and was therefore excluded from subsequent *in vivo* evaluations. In contrast, SAP100 significantly prolonged tail flick latency, whereas SAP200 reached the cutoff value of 8 s. Because SAP200 reached the predefined cutoff time, further increases in withdrawal latency could not be distinguished. The 0.4% lidocaine group also significantly increased withdrawal latency, although the difference from SAP200 could not be quantitatively distinguished because SAP200 reached the cutoff time. Acute analgesic effects under inflammatory pain conditions were further assessed using the hot plate test (Figure 3B). The sham group exhibited a normal withdrawal threshold of approximately 12 s, whereas CFA injection significantly reduced latency, confirming thermal hyperalgesia induced by inflammation. SAP100 and SAP200 did not significantly alter thermal response latency compared with the CFA group in the hot‐plate test. In contrast, lidocaine significantly increased latency relative to the CFA group. The sustained antinociceptive effects of SAP were evaluated using the von Frey test (Figure 3C). Before injection (0 min), paw withdrawal thresholds were comparable among the groups. Following injection, the CFA group exhibited reduced withdrawal thresholds throughout the observation period (5–120 min), indicating persistent mechanical hypersensitivity. SAP100 significantly increased the withdrawal threshold at 5 and 30 min, but the effect gradually declined from 60 min onward. SAP200 produced significant and sustained antinociceptive effects from 5 to 120 min, with significance maintained at 120 min. Although the absolute withdrawal threshold values were slightly lower than those of the lidocaine group, the duration of action was comparable.

To investigate whether these behavioral effects were associated with modulation of nociceptive neuronal activity, CGRP and IB4 expression in DRG tissues was analyzed by immunofluorescence staining. Representative images showed that CFA markedly increased CGRP immunoreactivity compared with the sham group, whereas SAP reduced CGRP‐positive neuronal signals in the SAP groups (Figure 3D). Quantitative analysis confirmed a significant increase in the proportion of CGRP‐positive neurons in the CFA group compared with the sham group (Figure 3E). SAP100 showed a nonsignificant decreasing trend, whereas SAP200 significantly reduced CGRP expression. Lidocaine also significantly suppressed CGRP expression, with no significant difference compared with SAP200. Similarly, CFA markedly increased IB4‐positive neuronal staining in DRG tissues, whereas SAP attenuated this response (Figure 3F). Quantification revealed that the proportion of IB4‐positive neurons was significantly elevated in the CFA group compared with sham. Both SAP100 and SAP200 significantly reduced IB4 expression, although no clear dose‐dependent difference was observed between the two doses. Lidocaine also significantly decreased IB4 expression, and no significant difference was detected compared with SAP200.

### 3.5. SAP Attenuates Microglial Activation and Inflammatory Gene Expression in Spinal Cord

**FIGURE 4:**
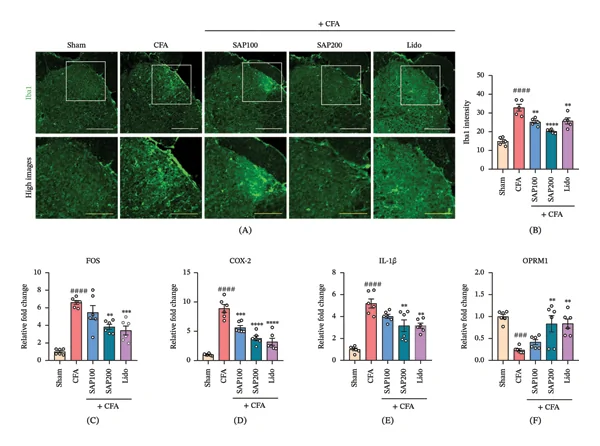
Effects of SAP on spinal Iba1 immunoreactivity and pain‐ and inflammation‐related gene expression. (A) Representative low‐ and high‐magnification immunofluorescence images of Iba1 staining in lumbar spinal cord tissues. The white scale bar = 100 μm, and the yellow scale bar = 50 μm. (B) Quantitative analysis of Iba1 fluorescence intensity in spinal cord sections (*n* = 5). (C, D) Relative mRNA expression levels of *FOS*, *COX-2*, *IL-1β*, and *OPRM1* in spinal cord tissues, as determined by quantitative real‐time PCR (*n* = 6). Data are presented as mean ± SEM. Statistical significance was analyzed by one‐way ANOVA followed by appropriate post hoc tests. ^###^ *p* < 0.001 and ^####^ *p* < 0.0001 versus sham group; ^∗∗^ *p* < 0.01, ^∗∗∗^ *p* < 0.001, and ^∗∗∗∗^ *p* < 0.0001 versus CFA group.

To investigate the effects of SAP on microglial activation *in vivo*, Iba1 immunostaining was performed on spinal cord tissues (Figure 4A). Iba1 fluorescence intensity was markedly increased in the CFA group compared with the sham group. SAP significantly attenuated this increase, with a greater reduction observed in the SAP200 group. Lidocaine injection also significantly decreased Iba1 intensity relative to the CFA group (Figure 4B). To further assess the effects of SAP on the expression of inflammation‐related genes, qPCR was performed. The mRNA expression levels of *FOS*, *COX-2*, and *IL-1β* were significantly upregulated in the CFA group compared to those in the sham group (Figure 4C). SAP significantly reduced the expression of these genes, with a more pronounced effect observed in the SAP200 group. Lidocaine injection significantly reduced the expression of these genes. In contrast, the expression of *OPRM1* was significantly lower in the CFA group than in the sham group (Figure 4D). SAP200 significantly increased *OPRM1* expression compared with the CFA group, whereas SAP100 showed a partial but nonsignificant increase. Lidocaine treatment also significantly restored *OPRM1* expression.

## 4. Discussion

The present study demonstrates that SAP exerts multimodal pain‐modulating effects associated with neuroprotection, local anesthetic‐like activity, and antinociceptive effects. Using complementary *in vitro* and *in vivo* models, SAP protected primary DRG neurons against H_2_O_2_‐induced oxidative injury, reduced nociceptive neuronal markers and neuropeptide release, attenuated CFA‐induced pain hypersensitivity, and suppressed neuroinflammatory responses in DRG and spinal cord tissues. These findings support the concept that SAP may provide broader pain‐modulating effects than simple symptomatic anesthesia.

Oxidative stress is a key contributor to neuronal sensitization and chronic pain progression. ROS can impair neurite integrity, alter ion channel excitability, and amplify inflammatory signaling in sensory neurons [20, 21]. In the present study, H_2_O_2_ exposure markedly reduced DRG neuronal viability, suppressed neurite outgrowth, and increased intracellular ROS levels, whereas SAP significantly reversed these changes. These findings suggest that SAP preserves sensory neuron structure and function under oxidative stress conditions. Eugenol, the principal bioactive constituent of clove, has been reported to possess antioxidant and cytoprotective properties through free‐radical scavenging and suppression of lipid peroxidation [18, 22]. Although eugenol is considered the principal bioactive constituent of clove and is likely responsible for a substantial proportion of the observed pharmacological effects, SAP is a complex botanical formulation containing multiple phytochemical constituents. Therefore, the present findings do not allow the biological activities of SAP to be attributed exclusively to eugenol. Other constituents may also contribute to the observed antioxidant and analgesic effects through additive or synergistic interactions, although their individual contributions remain to be determined. Future studies employing purified compounds, fractionated preparations, or component‐specific analyses will be necessary to clarify the relative contribution of each constituent.

Beyond general neuroprotection, SAP reduced several markers associated with nociceptive neuronal phenotypes, including IB4‐positive and CGRP‐positive neurons, as well as substance P release. IB4‐positive neurons largely represent nonpeptidergic nociceptors, whereas CGRP and substance P are classical pronociceptive neuropeptides involved in peripheral sensitization and neurogenic inflammation [23, 24]. The reduction of these markers suggests that SAP may be associated with reduced sensory neuron hyperexcitability rather than merely masking pain perception. Previous studies have shown that systemic eugenol decreases spinal levels of substance P and CGRP in osteoarthritis models and alleviates neuropathic or inflammatory pain behaviors [25, 26]. These findings are relevant to previous reports and suggest that clove‐derived pharmacopuncture may influence nociceptive signaling pathways involving DRG sensory neurons.

One of the most notable findings of this study was that SAP significantly prolonged withdrawal latency in the tail flick test following local administration. The high‐dose SAP group reached the predefined cutoff time and produced an antinociceptive response comparable to that observed with 0.4% lidocaine under the present experimental conditions. The tail flick assay is a well‐established spinal reflex‐based nociceptive test that is widely used to evaluate antinociceptive activity. In the present study, SAP was compared with lidocaine as a positive control because the objective was to evaluate its potential local anesthetic activity following local administration. However, because no centrally acting analgesic comparator (e.g., morphine) was included, the present findings do not allow definitive distinction between local anesthetic and analgesic mechanisms. Nevertheless, because SAP was administered locally and directly compared with lidocaine under identical experimental conditions, the results suggest that SAP possesses local anesthetic‐like activity. This observation is biologically plausible, as eugenol, a principal bioactive constituent of clove, has been reported to inhibit VGSCs, which are critical for action potential initiation and propagation in nociceptors [16]. Previous electrophysiological studies demonstrated that eugenol suppresses Na^+^ currents in DRG neurons through mechanisms distinct from lidocaine, including modulation of channel activation and inactivation states. In addition, studies in trigeminal sensory neurons have shown reversible inhibition of VGSC‐mediated excitability by eugenol [27]. Although previous studies strongly support a role for eugenol in VGSC inhibition, the present study did not directly compare purified eugenol with SAP. Therefore, the possibility that additional constituents present in SAP contribute to the observed local anesthetic‐like activity cannot be excluded.

CFA is a well‐established model of persistent inflammatory pain that induces robust peripheral inflammation through activation of innate immune responses and sustained release of proinflammatory mediators, resulting in peripheral sensitization and subsequent central sensitization within the spinal cord. These pathological changes contribute to the development of both thermal hyperalgesia and mechanical allodynia and closely resemble several features of persistent inflammatory pain observed in clinical conditions [28]. To comprehensively evaluate the analgesic profile of SAP, complementary behavioral assays were employed. The hot‐plate and von Frey assays were employed to evaluate supraspinal thermal nociception and mechanical hypersensitivity, respectively. Together, these complementary assays enabled evaluation of distinct components of nociceptive processing [29]. Notably, the behavioral response to SAP differed between the tail flick and hot‐plate tests. Whereas SAP produced a pronounced effect in the tail flick test, its effect in the hot‐plate test did not reach statistical significance. This discrepancy may reflect differences in the neural pathways underlying these assays. The tail flick response is predominantly mediated by a spinal reflex and may therefore be more sensitive to the peripheral and spinal effects of locally administered SAP, including its local anesthetic‐like activity. In contrast, the hot‐plate response involves greater supraspinal integration of nociceptive signals. Thus, the limited effect observed in the hot‐plate test may indicate that the acute antinociceptive effects of SAP are more prominent at the peripheral and spinal levels than at the supraspinal level. However, because the present study did not directly investigate site‐specific mechanisms, this interpretation remains to be confirmed.

In the CFA‐induced inflammatory pain model, SAP increased thermal response latency without reaching statistical significance, whereas clearer effects were observed in the von Frey assay, where SAP significantly attenuated mechanical hypersensitivity. Notably, the high‐dose SAP group maintained antinociceptive activity for up to 120 min, suggesting sustained antinociceptive activity under inflammatory conditions. Similar acute inflammatory pain studies have reported that significant antinociceptive effects are primarily observed within the first 120 min after treatment, although behavioral assessments may be continued for longer time periods [30]. At the tissue level, SAP significantly reduced CFA‐induced increases in CGRP and IB4 expression in DRG neurons. These findings are consistent with the in vitro observations and suggest that SAP may attenuate inflammatory activation of peripheral nociceptors. Lidocaine also reduced these markers, indicating that decreased peripheral afferent input itself may contribute to normalization of nociceptive signaling. However, the prolonged behavioral effects together with modulation of neuronal markers raise the possibility that SAP may provide broader biological actions beyond transient sodium channel blockade alone. Another notable finding was restoration of *OPRM1* expression. The μ‐opioid receptor is a critical endogenous analgesic target, and inflammatory states often reduce opioid receptor signaling efficiency [31]. The high‐dose SAP group significantly increased *OPRM1* mRNA relative to the CFA group, suggesting that SAP may contribute to normalization of endogenous inhibitory pathways. However, the present study does not establish whether this increase resulted from a direct regulatory effect of SAP on *OPRM1* expression or occurred indirectly as a consequence of reduced inflammation and nociceptive signaling. Given that SAP attenuated inflammatory responses and reduced nociceptive markers, the restoration of *OPRM1* expression may reflect normalization of endogenous opioid signaling secondary to reduced inflammatory burden. Eugenol has previously been reported to involve opioid‐related mechanisms, as naloxone partially reversed its antinociceptive effects in acute pain models [26]. Future studies employing pathway‐specific approaches will be necessary to determine whether SAP directly modulates *OPRM1* expression or acts indirectly through anti‐inflammatory mechanisms.

While these findings highlight the potential of SAP, several limitations should be considered. First, although quantitative HPLC analysis confirmed the eugenol content used for formulation standardization, SAP remains a complex botanical preparation containing multiple phytochemical constituents. Additional characterization, including comprehensive phytochemical fingerprinting, osmolarity assessment, and long‐term stability evaluation, will further strengthen formulation standardization. Second, behavioral assessments were limited to acute experimental models and a 120‐min observation period. Although SAP200 maintained significant antinociceptive activity throughout this period, the duration of analgesic efficacy beyond 120 min was not evaluated. Future studies with extended observation periods will be necessary to define the full duration of SAP‐induced analgesia. In addition, SAP was administered immediately following CFA injection to evaluate its acute pharmacological effects during the development of inflammatory pain. Therefore, the present design does not clearly distinguish preventive effects from therapeutic effects on established inflammatory pain. Although injection volume was matched among the sham, SAP50, SAP100, and lidocaine groups, the SAP200 group received a larger injection volume; therefore, a contribution of injection volume to the observed effects cannot be completely excluded. Future studies employing post‐treatment paradigms together with vehicle‐controlled and volume‐matched experimental designs will further clarify the therapeutic efficacy of SAP. Third, molecular analyses were limited to selected markers and did not directly examine specific signaling pathways such as VGSCs, TRPV1, MAPK, or NF‐κB. Future studies employing electrophysiological approaches together with pathway‐specific molecular analyses will be necessary to clarify the precise mechanisms underlying the pain‐modulating and local anesthetic‐like effects of SAP. Fourth, only male rats were used in this study to minimize variability associated with estrous cycle‐related hormonal fluctuations that may influence pain sensitivity. Therefore, the present findings may not be directly generalizable to female animals. Future studies including both male and female animals will be necessary to evaluate potential sex‐related differences and confirm the broader applicability of SAP. In addition, a formal a priori sample‐size calculation was not performed. Although the sample sizes were consistent with previous studies using similar experimental models, future studies employing prospectively powered experimental designs would further strengthen the robustness of the findings. Finally, although the present formulation underwent GMP manufacturing and routine quality control, dedicated safety evaluations, including local tissue compatibility, injection‐site histopathology, systemic toxicity, and repeated‐dose safety, were not performed. Therefore, the present findings are insufficient to establish the safety of SAP as an injectable formulation, and comprehensive toxicological assessment is required before clinical translation can be considered.

## 5. Conclusion

In the present acute experimental models, SAP demonstrated multimodal pain‐modulating effects by protecting sensory neurons from oxidative injury, reducing nociceptive neuropeptides, increasing withdrawal latency following local administration, attenuating inflammatory hypersensitivity, and suppressing peripheral and central pain‐related signaling. Collectively, these findings provide experimental evidence supporting the antinociceptive and local anesthetic‐like potential of SAP. Furthermore, modulation of oxidative stress and nociceptive signaling may contribute, at least in part, to these pharmacological effects.

## Author Contributions

In‐Hyuk Ha: conceptualization, funding acquisition, project administration, resources, supervision, and writing–review and editing. Hyunseong Kim: data curation, formal analysis, investigation, methodology, software, visualization, and writing–original draft. Jin Young Hong: formal analysis, validation, visualization, and writing–review and editing. Changhwan Yeo: formal analysis, methodology, and validation. Wan‐Jin Jeon: formal analysis, methodology, and validation. Hyun Kim: formal analysis, methodology, and visualization. Junseon Lee: formal analysis and visualization. Yoon Jae Lee: validation and writing–review and editing. Seung Ho Baek: validation and writing–review and editing.

## Funding

This work was supported by the National Research Foundation of Korea grant funded by the Korea government (RS‐2025‐23323091).

## Disclosure

All authors read and approved the final manuscript.

## Ethics Statement

This study was approved by the Jaseng Animal Care and Use Committee (approval no. JSR‐2023‐02‐002‐A‐002).

## Conflicts of Interest

The authors declare no conflicts of interest.

## Supporting Information

Additional supporting information can be found online in the Supporting Information section.

## Supporting information


**Supporting Information** Supporting Figure S1. Preliminary optimization of the tail flick test, including determination of the thermal cutoff time and selection of the lidocaine concentration used as the positive control. Supporting Figure S2. HPLC‐based phytochemical characterization and quantitative standardization of SAP. Representative chromatograms of the authentic eugenol standard and SAP are shown together with UV spectral comparison, repeatability data from six independent injections, and quantitative determination of eugenol content.

## Data Availability

The data that support the findings of this study are available from the corresponding author upon reasonable request.
