# Supplementary material for: Analgesic and Local Anesthetic Effects of a Clove‐Derived Injectable Formulation via Modulation of Oxidative Stress and Nociceptive Signaling
Source: Pain Res Manag. 2026 Sep 20;2026:5004039. doi: 10.1155/prm/5004039 (PMC13591119; doi:10.1155/prm/5004039)
Supplement: Supplementary file 1 [file PRM-2026-5004039-s001.docx]

**Figure S1. Preliminary optimization of tail flick assay conditions.** (A) Baseline tail flick latency was approximately 2 s in untreated rats, whereas thermal exposure exceeding 10 s was associated with tissue injury. Therefore, the cut-off time was set at 8 s to ensure assay sensitivity while preventing thermal damage. (B) Lidocaine concentration screening demonstrated that 0.2% lidocaine produced insufficient anesthetic responses, whereas 0.5% lidocaine generated near-ceiling effects, with five of six animals showing no withdrawal response before the cut-off time and therefore being assigned the maximum latency value of 8 s. In contrast, 0.4% lidocaine produced stable and measurable prolongation of withdrawal latency without consistently reaching the cut-off threshold and was selected as the positive control for subsequent experiments.

**Figure S2. Phytochemical characterization and standardization of SAP.** (A) Representative HPLC chromatogram of the authentic eugenol reference standard. (B) Representative HPLC chromatogram of SAP showing a major peak corresponding to eugenol. (C) UV absorption spectrum of the eugenol reference standard. (D) UV absorption spectrum of the corresponding peak detected in SAP, confirming peak identity.

**Table S1. Repeatability of six independent SAP injections presented as retention time and peak area measurements.**

| **Injection** | **RT (min)** | **Peak Area** | **Peak Height** |
| --- | --- | --- | --- |
| 1 | 14.303 | 11404.877 | 1180.201 |
| 2 | 14.301 | 11233.471 | 1168.924 |
| 3 | 14.296 | 11166.800 | 1161.589 |
| 4 | 14.284 | 11403.994 | 1185.171 |
| 5 | 14.294 | 11466.295 | 1187.569 |
| 6 | 14.292 | 11416.297 | 1182.021 |

**Table S2. Quantitative determination of eugenol in SAP**

| **Parameter** | **Result** |
| --- | --- |
| Standard purity | 99.8% |
| Standard concentration | 0.998 μL/mL |
| Number of injections | 6 |
| Mean eugenol concentration | **1.029 ± 0.011 μL/mL** |
| CV (%) | 1.05 |
| Equivalent concentration used in the study | 6.875 mM |

**Table S3. RNA purity and concentration of spinal cord samples used for quantitative real-time PCR analysis.**

| Sample | 260 Raw | 280 Raw | 320 Raw | 260 | 280 | **260/280** | **ng/μL** |
| --- | --- | --- | --- | --- | --- | --- | --- |
| Sham_1 | 0.298 | 0.185 | 0.09 | 0.204 | 0.092 | **2.206** | **163.1** |
| Sham_2 | 0.162 | 0.105 | 0.059 | 0.099 | 0.044 | **2.262** | **78.9** |
| Sham_3 | 0.256 | 0.159 | 0.08 | 0.172 | 0.077 | **2.237** | **137.6** |
| Sham_4 | 0.216 | 0.124 | 0.048 | 0.165 | 0.074 | **2.227** | **131.9** |
| Sham_5 | 0.246 | 0.136 | 0.04 | 0.202 | 0.094 | **2.152** | **161.7** |
| Sham_6 | 0.113 | 0.076 | 0.046 | 0.063 | 0.027 | **2.303** | **50.5** |
| CFA_1 | 0.275 | 0.175 | 0.086 | 0.185 | 0.087 | **2.128** | **148.4** |
| CFA_2 | 0.318 | 0.193 | 0.08 | 0.234 | 0.11 | **2.124** | **187.0** |
| CFA_3 | 0.42 | 0.253 | 0.111 | 0.305 | 0.139 | **2.191** | **244.4** |
| CFA_4 | 0.471 | 0.301 | 0.169 | 0.298 | 0.13 | **2.299** | **238.6** |
| CFA_5 | 0.432 | 0.252 | 0.102 | 0.327 | 0.149 | **2.198** | **261.5** |
| CFA_6 | 0.12 | 0.078 | 0.041 | 0.076 | 0.035 | **2.153** | **60.6** |
| SA100_1 | 0.314 | 0.174 | 0.056 | 0.255 | 0.117 | **2.189** | **204.3** |
| SA100_2 | 0.301 | 0.162 | 0.043 | 0.255 | 0.117 | **2.175** | **204.1** |
| SA100_3 | 0.176 | 0.104 | 0.041 | 0.131 | 0.061 | **2.146** | **104.4** |
| SA100_4 | 0.382 | 0.199 | 0.043 | 0.335 | 0.154 | **2.182** | **268.2** |
| SA100_5 | 0.302 | 0.168 | 0.055 | 0.244 | 0.11 | **2.209** | **195.0** |
| SA100_6 | 0.159 | 0.098 | 0.043 | 0.113 | 0.053 | **2.137** | **90.1** |
| SA200_1 | 0.265 | 0.144 | 0.041 | 0.221 | 0.102 | **2.171** | **176.7** |
| SA200_2 | 0.365 | 0.191 | 0.04 | 0.321 | 0.149 | **2.163** | **257.2** |
| SA200_3 | 0.25 | 0.138 | 0.04 | 0.206 | 0.095 | **2.166** | **165.2** |
| SA200_4 | 0.183 | 0.106 | 0.039 | 0.141 | 0.065 | **2.161** | **112.4** |
| SA200_5 | 0.196 | 0.113 | 0.04 | 0.152 | 0.071 | **2.154** | **121.7** |
| SA200_6 | 0.124 | 0.08 | 0.041 | 0.078 | 0.037 | **2.148** | **62.8** |
| Lido_1 | 0.356 | 0.32 | 0.285 | 0.067 | 0.032 | **2.065** | **53.2** |
| Lido_2 | 0.104 | 0.075 | 0.056 | 0.044 | 0.017 | **2.592** | **35.1** |
| Lido_3 | 0.212 | 0.121 | 0.041 | 0.167 | 0.078 | **2.154** | **133.7** |
| Lido_4 | 0.123 | 0.079 | 0.04 | 0.08 | 0.037 | **2.172** | **64.0** |
| Lido_5 | 0.27 | 0.148 | 0.041 | 0.225 | 0.104 | **2.158** | **179.9** |
| Lido_6 | 0.168 | 0.122 | 0.082 | 0.083 | 0.038 | **2.168** | **66.1** |
